# HiRo-SLAM: A High-Accuracy and Robust Visual-Inertial SLAM System with Precise Camera Projection Modeling and Adaptive Feature Selection

**DOI:** 10.3390/s26020711

**Published:** 2026-01-21

**Authors:** Yujuan Deng, Liang Tian, Xiaohui Hou, Xin Liu, Yonggang Wang, Xingchao Liu, Chunyuan Liao

**Affiliations:** 1 School of Mathematical Sciences, Hebei Normal University, Shijiazhuang 050024, China; 2 College of Future Information Technology, Shijiazhuang University, Shijiazhuang 050035, China; 3 Hebei Internet of Things Intelligent Perception and Application Technology Innovation Center, Shijiazhuang 050035, China; 4 Information Technology Center, Hebei Normal University, Shijiazhuang 050024, China; 5 HiScene Information Technology Co., Ltd., Shanghai 201203, China; 6 School of Information Technology and Cyber Security, People’s Public Security University of China, Beijing 100038, China

**Keywords:** visual-inertial SLAM, camera projection model, analytical Jacobian, adaptive feature selection, Graduated Non-Convexity, Point-Line Fusion, robustness

## Abstract

**Highlights:**

**What are the main findings?**

- We introduce a unified optimization framework that integrates a precise camera projection model (incorporating analytical distortion Jacobians) with Graduated Non-Convexity (GNC) robust estimation. This approach significantly improves system accuracy and stability by simultaneously minimizing error sources and optimizing the backend.
- The Visibility Pyramid-based Adaptive Non-Maximum Suppression (P-ANMS) mechanism, combined with a hybrid point-line frontend fusing XFeat and SOLD2, addresses core challenges in feature tracking. This integration is particularly effective in environments characterized by weak textures or repetitive structures.

**What are the implications of the main findings?**

- Comprehensive validation demonstrates that HiRo-SLAM achieves superior positioning accuracy and scene adaptability across multiple benchmarks, setting a new state-of-the-art performance standard.
- The integration of precise camera modeling, adaptive feature selection, and robust optimization offers a comprehensive technical solution for deploying high-precision visual-inertial SLAM systems in complex real-world environments.

**Abstract:**

HiRo-SLAM is a visual-inertial SLAM system developed to achieve high accuracy and enhanced robustness. To address critical limitations of conventional methods, including systematic biases from imperfect camera models, uneven spatial feature distribution, and the impact of outliers, we propose a unified optimization framework that integrates four key innovations. First, Precise Camera Projection Modeling (PCPM) embeds a fully differentiable camera model in nonlinear optimization, ensuring accurate handling of camera intrinsics and distortion to prevent error accumulation. Second, Visibility Pyramid-based Adaptive Non-Maximum Suppression (P-ANMS) quantifies feature point contribution through a multi-scale pyramid, providing uniform visual constraints in weakly textured or repetitive regions. Third, Robust Optimization Using Graduated Non-Convexity (GNC) suppresses outliers through dynamic weighting, preventing convergence to local minima. Finally, the Point-Line Feature Fusion Frontend combines XFeat point features with SOLD2 line features, leveraging multiple geometric primitives to improve perception in challenging environments, such as those with weak textures or repetitive structures. Comprehensive evaluations on the EuRoC MAV, TUM-VI, and OIVIO benchmarks show that HiRo-SLAM outperforms state-of-the-art visual-inertial SLAM methods. On the EuRoC MAV dataset, HiRo-SLAM achieves a 30.0% reduction in absolute trajectory error compared to strong baselines and attains millimeter-level accuracy on specific sequences under controlled conditions. However, while HiRo-SLAM demonstrates state-of-the-art performance in scenarios with moderate texture and minimal motion blur, its effectiveness may be reduced in highly dynamic environments with severe motion blur or extreme lighting conditions.

## 1. Introduction

Visual-Inertial SLAM (VINS) is essential for autonomous navigation in GPS-denied environments, with applications in robotics, UAVs, augmented reality, and mobile systems [1]. While recent systems like ORB-SLAM3 [2] and VINS-Mono [3] perform well in structured environments, they face significant challenges in unconstrained real-world settings. These challenges stem from three primary factors: inaccurate camera models, insufficient robustness in dynamic environments, and perceptual limitations, particularly in feature-sparse or repetitive environments.

### 1.1. Inaccurate Camera Models

Many VINS frameworks simplify camera models by neglecting distortion parameters, assuming their impact is minimal [2,3]. However, even small errors can accumulate over time, leading to significant drift. Methods like DeepVINS [4] attempt to learn camera parameters, but they are often overfitted to specific datasets, reducing generalization. Although frameworks like OpenVINS [5] use efficient optimization techniques, such as the First Estimate Jacobian (FEJ) method to reduce computational errors, their conservative optimization strategies still limit performance improvements. Unlike ORB-SLAM3 [2], which uses simplified camera models, HiRo-SLAM integrates Precise Camera Projection Modeling (PCPM), embedding full distortion parameters into the optimization process. This ensures long-term pose estimation accuracy and prevents error accumulation, offering a major improvement over traditional methods that omit or approximate camera models.

### 1.2. Perceptual Limitations in Feature-Sparse or Repetitive Environments

VINS systems often struggle with two perceptual issues: spatial organization and feature diversity. The first issue stems from the reliance of traditional methods [5,6] on techniques such as non-maximum suppression (NMS) [7] and bucketing [8]. These methods struggle to maintain a globally uniform feature distribution, leading to tracking instability, especially in feature-sparse environments. HiRo-SLAM overcomes these issues with Visibility Pyramid-based Adaptive Non-Maximum Suppression (P-ANMS) [9], which ensures a uniform feature distribution by evaluating spatial contribution across multiple scales. This method improves feature coverage even in challenging environments.

The second challenge is feature diversity. When relying solely on point features, systems experience “feature deprivation” in low-texture or repetitive environments. While methods like LSD-SLAM [10] have incorporated line features [11,12], traditional line extraction methods (e.g., LSD [13], LBD [14]) are still vulnerable to noise and lighting changes, making them unreliable in dynamic environments. For feature diversity, HiRo-SLAM integrates a point-line feature fusion frontend, combining XFeat point features [15] with robust SOLD2 line features [16]. This fusion enhances stability in weakly textured and repetitive regions, where point-based systems struggle.

### 1.3. Insufficient Robustness in Dynamic Environments

Outliers from dynamic environments, such as moving objects or sudden illumination changes, are common in VINS. While M-estimators [17] can handle moderate outliers, their performance deteriorates under high outlier conditions, often causing trajectory divergence. Methods like R-VINS [18] improve robustness but still struggle with extreme outliers. HiRo-SLAM addresses this challenge with Graduated Non-Convexity (GNC) [19], which dynamically adjusts the cost function to suppress outliers, ensuring stable convergence even in challenging conditions. This outperforms existing methods that rely on fixed thresholds for outlier rejection.

To address these challenges, we propose HiRo-SLAM, a visual-inertial SLAM system with four key innovations:

(1) Precise Camera Projection Modeling (PCPM): This approach integrates full distortion parameters into the optimization process, which improves pose estimation accuracy and prevents error accumulation.
(2) Visibility Pyramid-based Adaptive Non-Maximum Suppression (P-ANMS): By enhancing feature spatial distribution, P-ANMS ensures more reliable pose estimation, especially in feature-sparse regions.
(3) Integration of Graduated Non-Convexity (GNC) in the VINS Backend: GNC enhances robustness to outliers, providing stable optimization even in dynamic and unpredictable environments.
(4) Lightweight Point-Line Feature Fusion Frontend: The fusion of XFeat point features with SOLD2 line features strengthens robustness in environments with low texture or repetitive patterns.

## 2. Related Work

### 2.1. Visual-Inertial SLAM System Architecture

The development of visual-inertial SLAM (VINS) has shifted from filtering-based methods to optimization-based approaches. While filtering methods like MSCKF [20] offer computational efficiency, they are limited by linearization errors. This constraint has led to the prevalence of nonlinear optimization in modern systems. Notable examples such as OKVIS [21] and VINS-Mono [3] have advanced the field through tightly coupled sliding window optimization. ORB-SLAM3 [2] further improved upon this by introducing a multi-map system and enhanced relocalization, setting the current state-of-the-art for feature-based VINS. Despite these advancements, these systems still rely on static camera parameters and exhibit insufficient robustness in both backend estimation and frontend feature management. These limitations directly motivate our work.

### 2.2. Camera Modeling and Precise Projection

Accurate camera modeling is crucial for reliable VINS performance. Common models, such as Brown–Conrady [22] for conventional lenses and Kannala–Brandt [23] for fisheye lenses, are widely used to handle lens distortion. However, many VINS methods simplify these models or neglect distortion parameters entirely during nonlinear optimization. For instance, OpenVINS [5] incorporates online calibration by jointly estimating sensor parameters in real-time but does so with conservative update policies that limit potential improvements in accuracy. Furthermore, while many photometric approaches like DSO [24] implicitly handle distortion in their error formulation, they often sacrifice geometric interpretability. Many VINS methods, due to computational overhead and real-time constraints, either simplify optimization models or rely on numerical methods. As a result, the gradient computations may be imprecise, leading to suboptimal optimization performance. In contrast, our work offers a more precise analytical framework by directly integrating camera parameter optimization into nonlinear pose estimation. This is achieved through derived Jacobians, which allow for greater accuracy while maintaining full geometric transparency. Our method provides a more interpretable and effective solution for pose estimation in VINS.

### 2.3. Feature Selection and Distribution Optimization

The spatial distribution of visual features is crucial for VINS stability. Detectors like FAST [25] and ORB [26] tend to generate clustered features, while traditional distribution strategies, such as non-maximum suppression (NMS) and bucketing, fail to ensure globally optimal feature placement [27]. Adaptive NMS (ANMS) partially addresses this by balancing inter-feature distance with response strength, improving feature distribution uniformity. However, ANMS operates primarily within a local context, lacking a global assessment of spatial coverage. To address this, we incorporate a multi-scale visibility pyramid [28] into the feature management process, providing a global perspective that improves feature selection. Our key contribution is integrating this visibility pyramid-based adaptive selection mechanism directly into the SLAM frontend, rather than treating it as a separate preprocessing step.

### 2.4. Robust Estimation Theory

Robust outlier mitigation is essential for reliable state estimation. Traditional M-estimators [17], such as the Huber norm, maintain computational efficiency but perform poorly when outlier ratios exceed established thresholds (typically over 30% [29]). The Graduated Non-Convexity (GNC) framework [19,30] offers a mathematically grounded solution for high-outlier scenarios, progressively transforming convex cost functions into non-convex ones, demonstrating strong performance in spatial perception tasks [31]. However, GNC has not been systematically incorporated into tightly coupled VINS backends, a gap that our work directly addresses.

### 2.5. Point, Line and Multi-Geometric Feature Fusion

Integrating multiple geometric primitives—particularly points and lines—has become critical for overcoming the limitations of single-feature representations in challenging environments [10,11]. The development of line segment extraction methods has evolved from conventional approaches like LSD [12] to more recent learned methods, such as LBD [13], LETR [32], and SOLD2 [16], with continuous improvements in feature detection and association. In parallel, point feature detectors like SuperPoint [33] and recent developments like XFeat [15] and ALIKE [34] have significantly improved computational efficiency and feature matching density. However, integrating and managing heterogeneous features effectively within SLAM frontends remains a challenge, particularly for real-time performance and system integration. To overcome this, we propose a lightweight point-line feature fusion framework that combines XFeat [15] and SOLD2 [16], achieving improved feature diversity and system robustness without compromising computational efficiency.

## 3. Methodology

### 3.1. System Architecture

**Figure 1:**
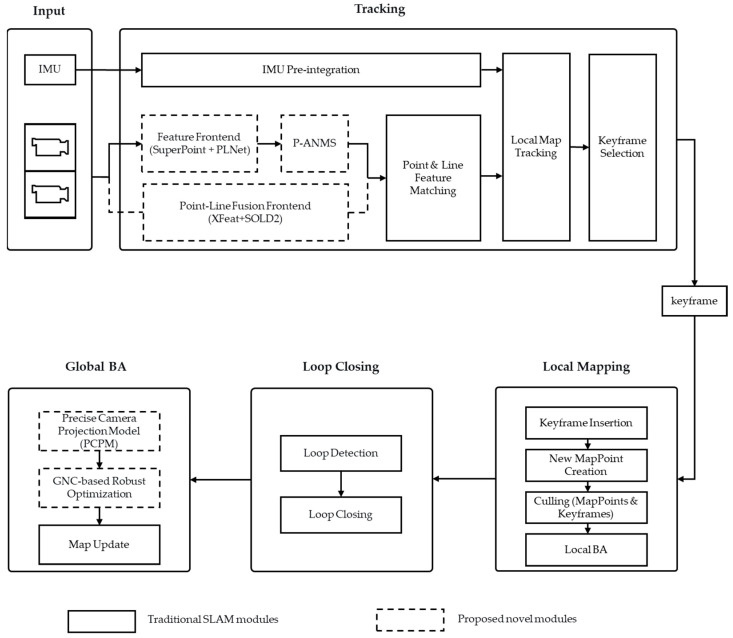
The HiRo-SLAM framework includes tracking, local mapping, and global bundle adjustment (BA) & loop closing for robust SLAM. The feature extraction module can use either the SuperPoint + PLNet implementation or the proposed Point-Line Fusion Frontend (XFeat + SOLD2), depending on the environmental characteristics.

HiRo-SLAM introduces a unified and adaptive framework for tightly coupled visual-inertial SLAM. As shown in Figure 1, the system architecture integrates four core innovations that synergistically address key limitations of conventional VINS systems. These limitations include errors from inaccurate camera models, sensitivity to outliers, and constrained feature perception capabilities. The innovations are: Precise Camera Projection Modeling (incorporating analytical distortion Jacobians), Visibility Pyramid-based Adaptive Non-Maximum Suppression (P-ANMS), GNC-based Robust Estimation, and a Point-Line Feature Fusion Frontend.

HiRo-SLAM adopts the SuperPoint + PLNet architecture as the default frontend configuration. This setup serves as the baseline for our primary evaluations, including the EuRoC benchmark detailed in Section 4.1. To further address perceptual degradation in extreme environments—specifically those characterized by weak textures or repetitive structures—we introduce an optional Point-Line Fusion Frontend (integrating XFeat and SOLD2). This specialized module serves as a robust alternative to the default frontend, designed to maintain tracking stability in scenarios where traditional point-based features are prone to failure. The frontend is responsible for feature extraction and matching, providing a set of 3D points and keyframes. Based on these data, the backend performs bundle adjustment to achieve precise estimation of camera poses and the 3D structure.

### 3.2. Precise Camera Projection Modeling (PCPM) with Analytical Distortion Jacobians for Enhanced Online Optimization

Many methods, even when using offline-calibrated camera parameters, simplify the distortion model during nonlinear optimization or rely on numerical approximations for Jacobian computation. This simplification leads to systematic errors in the reprojection error gradients, which propagate through camera pose and 3D landmark optimization. As a result, the overall accuracy and robustness of the SLAM system are compromised, especially in regions of high image distortion or near the image periphery where such simplifications are more problematic.

To address this issue, we propose a rigorous analytical approach to camera projection modeling, even when distortion parameters are predefined. Our key contribution is the explicit derivation of distortion parameter propagation, which allows for the construction of a precise analytical Jacobian matrix. This integration ensures that the camera model used in optimization is fully differentiable, enabling precise gradient computation and mitigating systematic error accumulation during state optimization, such as pose and 3D landmarks.

Given a 3D point $P_{w}$ in the world coordinate system, its projection onto the normalized image plane yields coordinates (x,y). After distortion correction, these coordinates are transformed according to the standard plumb-bob model:

(1) $$\begin{array}{cc} x_{d} & =x(1+k_{1}r^{2}+k_{2}r^{4})+2p_{1}xy+p_{2}(r^{2}+2x^{2})\\ y_{d} & =y(1+k_{1}r^{2}+k_{2}r^{4})+p_{1}(r^{2}+2y^{2})+2p_{2}xy \end{array}$$

where $r^{2}=x^{2}+y^{2}$, $k_{1}$ and $k_{2}$ are the radial distortion coefficients, and $p_{1}$ and $p_{2}$ are the tangential distortion coefficients.

Next, transformation to pixel coordinates via the camera intrinsic matrix gives:

(2) $$z^{\mathrm{pred}}=\left[\begin{array}{c} u\\ v \end{array}\right]=\left[\begin{array}{c} f_{x}x_{d}+c_{x}\\ f_{y}y_{d}+c_{y} \end{array}\right]$$

where $f_{x}$ and $f_{y}$ are the focal lengths in pixel units, and $c_{x}$ and $c_{y}$ denote the principal point coordinates.

The reprojection error is defined as the difference between the observed and predicted pixel coordinates:

(3) $$e=z^{\mathrm{obs}}-z^{\mathrm{pred}}$$

where $z^{\mathrm{obs}}$ corresponds to the observed pixel coordinates.

A distinctive feature of our system is the derivation of the analytical Jacobian matrix, which incorporates the full set of fixed distortion parameters to compute the derivatives of the reprojection error with respect to the optimized states. This overall Jacobian is expressed as:

(4) $$J=\frac{\partial e}{\partial x}=-J_{I}\cdot J_{P}\cdot J_{T}$$

Here, $J_{P}$ and $J_{T}$ correspond to the derivatives of normalized coordinates with respect to camera coordinates, and camera coordinates with respect to system states, respectively.

The Projection Jacobian $J_{P}$ characterizes the differential relationship between a 3D point in the camera coordinate system and its corresponding normalized image coordinates:

(5) $$J_{P}=\frac{\partial p_{n}}{\partial P_{c}}=\left[\begin{array}{ccc} \frac{1}{Z_{c}} & 0 & -\frac{X_{c}}{Z_{c}^{2}}\\ 0 & \frac{1}{Z_{c}} & -\frac{Y_{c}}{Z_{c}^{2}} \end{array}\right]$$

The Transformation Jacobian $J_{T}$ captures the differential relationship between camera coordinates $P_{c}$ and system states x:

(6) $$J_{T}=\left[\frac{\partial P_{c}}{\partial x}\right]$$

Finally, the core term $J_{I}$ characterizes the differential relationship between pixel coordinates and normalized distorted coordinates:

(7) $$J_{I}=\left[\begin{array}{cc} \frac{\partial u}{\partial x} & \frac{\partial u}{\partial y}\\ \frac{\partial v}{\partial x} & \frac{\partial v}{\partial y} \end{array}\right]=\left[\begin{array}{cc} f_{x}\frac{\partial x_{d}}{\partial x} & f_{x}\frac{\partial x_{d}}{\partial y}\\ f_{y}\frac{\partial y_{d}}{\partial x} & f_{y}\frac{\partial y_{d}}{\partial y} \end{array}\right]$$

The partial derivatives are analytically expressed as:

(8) $$\begin{array}{cc} \frac{\partial x_{d}}{\partial x} & =(1+k_{1}r^{2}+k_{2}r^{4})+2k_{1}x^{2}+4k_{2}r^{2}x^{2}+2p_{1}y+6p_{2}x\\ \frac{\partial x_{d}}{\partial y} & =2k_{1}xy+4k_{2}r^{2}xy+2p_{1}x+2p_{2}y\\ \frac{\partial y_{d}}{\partial x} & =2k_{1}xy+4k_{2}r^{2}xy+2p_{1}x+2p_{2}y\\ \frac{\partial y_{d}}{\partial y} & =(1+k_{1}r^{2}+k_{2}r^{4})+2k_{1}y^{2}+4k_{2}r^{2}y^{2}+6p_{1}y+2p_{2}x \end{array}$$

This analytical formulation fully captures the nonlinear characteristics of the camera projection model, including predefined distortion parameters. By utilizing precise analytical derivatives, our system ensures that reprojection errors are accurately propagated back to the optimized system states (camera poses and 3D landmarks). This approach mitigates systematic errors in gradient computation, which would otherwise arise from simplified distortion models or numerical approximations. As shown in the ablation studies (Section 4.5), the incorporation of Precise Camera Projection Modeling (PCPM) contributes 19.67% to the total performance improvement, highlighting its critical role in reducing error accumulation and enhancing pose estimation accuracy, especially in challenging environments with significant distortion.

### 3.3. P-ANMS: Adaptive Uniform Feature Extraction via Visibility Pyramid

Feature point extraction plays a crucial role in visual SLAM systems, but its limitations become evident in challenging environments. Non-Maximum Suppression (NMS), while effective for ensuring feature distinctiveness and strong local responses, suffers from aggregation of feature points in texture-rich regions. Conversely, in texture-less or repetitive environments (e.g., large walls, floors, or metallic surfaces), NMS leaves large blank areas, significantly increasing the risk of feature tracking and matching failures. Adaptive Non-Maximum Suppression (ANMS) improves feature distribution uniformity by incorporating a local distance constraint. However, ANMS relies only on local neighborhood information to determine suppression radii, lacking a global assessment of spatial coverage. This limits its ability to adequately fill sparse regions and achieve an overall balanced feature distribution.

To overcome these challenges, we introduce Visibility Pyramid-based Adaptive Non-Maximum Suppression (P-ANMS). P-ANMS aims to achieve a superior, globally uniform distribution of feature points while maintaining high-quality responses. Building on the local distance constraint of ANMS, our method introduces a multi-scale Visibility Pyramid to assess the contribution of candidate feature points to spatial uniformity. The goal is to prioritize feature points that effectively cover sparse or blank regions within the image, thereby providing more robust and comprehensive visual constraints for pose estimation and map building.

The P-ANMS algorithm proceeds as follows:

1. Initial Sorting: We begin by sorting the candidate feature points $C=\{c_{i}=(x_{i},y_{i})\}$ and their corresponding response scores $\left\{s_{i}\right\}$ in descending order based on the response score $s_{i}$. The response score $s_{i}$ refers to the confidence or strength of the feature point, which is determined by the activation of the feature detector (e.g., SuperPoint). A higher score indicates a more distinctive and reliable feature point for matching. This ensures that higher-response features are prioritized when evaluating their distribution contribution.
2. Visibility Pyramid Construction and Distribution Score Calculation:

Visibility Pyramid Model: The image is divided into L different scales. At each level l(l∈[0,L−1]), the image is uniformly partitioned into $2^{l}\times 2^{l}$ grid cells.

Distribution Score Calculation: For each candidate point $c_{i}$ in the sorted list, we compute its Visibility Pyramid Distribution Score $S_{i}$. This score quantifies the potential contribution of $c_{i}$ to improving the spatial uniformity of the selected feature set. The score is calculated as:

(9) $$S_{i}=\sum_{l=0}^{L-1}w_{l}\cdot N_{l}(c_{i},\mathrm{SelectedSet})\;$$

where $N_{l}(c_{i},\mathrm{SelectedSet})$ evaluates the increase in the number of non-empty grid cells at pyramid level l if $c_{i}$ is added to the selected set. The weight $w_{l}$ typically increases with l, emphasizing the importance of filling sparse regions at finer scales. A higher $S_{i}$ indicates that $c_{i}$ will significantly improve feature distribution by occupying under-represented areas across multiple scales.

3. Final Selection: All candidate points, paired with their computed $\left(c_{i},S_{i}\right)$ scores, are collected. These points are then sorted in descending order based on $S_{i}$. Finally, the top N points from this reordered list are selected as the final set of uniformly distributed feature points. This two-stage sorting strategy ensures that the selected features have both strong response strengths and maximum spatial coverage within the image.

The underlying rationale of this method is that $S_{i}$ precisely quantifies how much a candidate point $c_{i}$ contributes to the distribution uniformity of the current feature set. If a candidate point falls into a sparse region, its inclusion will significantly increase the number of non-empty grid cells across various pyramid levels, resulting in a high $S_{i}$. In contrast, if the point is in an already dense area, its score will be lower. This mechanism allows P-ANMS to actively identify and populate blank areas, optimizing for the goal of achieving maximum distribution uniformity for a fixed number of points.

The proposed visual-inertial SLAM framework incorporates an adaptive feature selection strategy, where key parameters significantly affect its performance. The visibility pyramid levels determine the number of levels in the pyramid, which influences the scale at which it operates. Meanwhile, the keypoint threshold defines the minimum response score required for a keypoint to be considered. A higher threshold reduces the number of detected keypoints.

To ensure reproducibility and validate the advantages of P-ANMS, we analyze the V2_03_difficult sequence from the EuRoC MAV dataset, recorded in a Vicon motion capture room. This compact environment imposes severe challenges on algorithmic robustness. Images were captured using a VI-Sensor (model MT9M034; ON Semiconductor, Phoenix, AZ, USA) at 20 Hz with a resolution of 752×480. The selected frames feature low-reflectance surfaces, such as dark curtains and concrete, which create regions of low-contrast texture. Under these conditions, where local gradient information significantly degrades, the performance advantages of P-ANMS are most evident.

**Figure 2:**
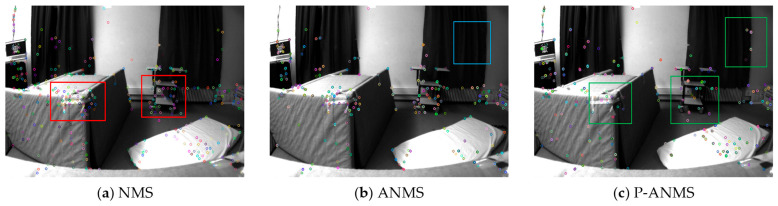
Comparison of feature distribution among NMS, ANMS, and P-ANMS strategies in the EuRoC V2_03_difficult sequence. (Red, blue, and green boxes denote regions of feature overcrowding, coverage gaps, and uniform distribution, respectively).

Figure 2 highlights the distinct differences in feature distribution:

As shown in Figure 2a, NMS leads to excessive feature clustering within the red boxes, causing significant local redundancy. While ANMS (Figure 2b) partially mitigates this clustering, it fails to achieve ideal global uniformity. Specifically, the curtain area (blue box) exhibits noticeable feature voids. In contrast, P-ANMS (Figure 2c) demonstrates superior distribution characteristics. As highlighted in the green boxes, features on the cart and curtains are more evenly dispersed. This approach effectively eliminates local redundancy while ensuring robust coverage across critical regions.

These results indicate that P-ANMS optimizes the spatial distribution of features, providing a more stable and reliable foundation for subsequent pose estimation in SLAM.

This visual improvement translates directly into enhanced localization accuracy. As shown in the ablation studies (Section 4.5), P-ANMS reduces the average RMSE from 0.0245 to 0.0212, contributing 10.82% to the overall performance gain. The impact is particularly pronounced in the V2_03_difficult sequence of the EuRoC dataset, where the RMSE drops from 0.0366 to 0.0253. These results demonstrate that P-ANMS effectively mitigates tracking and matching failures in challenging environments characterized by texture-less areas and motion blur.

Beyond accuracy, the robustness of P-ANMS is further validated through parameter ablation experiments in Section 4.6. These tests highlight the influence of key parameters, such as the keypoint threshold and pyramid levels, on system performance. Our findings reveal that P-ANMS maintains stable performance across a reasonable parameter range, providing empirical evidence for the method’s reliability in diverse conditions.

### 3.4. Robust Optimization and Outlier Rejection Based on Graduated Non-Convexity (GNC)

Traditional visual SLAM backends typically use fixed robust kernel functions, such as Huber or Cauchy, during nonlinear optimization to reduce the influence of outliers on system state estimation. However, these kernel functions are inherently convex or approximately convex. In scenarios with a high proportion of outliers or complex environments, they often converge to local minima, reducing both accuracy and robustness. Additionally, conventional outlier rejection methods often require alternating between optimization and manual outlier handling, which is inefficient and cannot guarantee global optimality.

To address these limitations, we introduce Graduated Non-Convexity (GNC) to enhance the robustness of backend optimization. Unlike traditional two-stage approaches, the core idea of GNC is to progressively transform the original non-convex optimization problem into a series of convex subproblems using a continuous convex approximation process. The key innovation of GNC lies in its dynamic weighting mechanism: initially, a larger non-convexity parameter μ is set, which ensures that the cost function remains convex, facilitating global convergence before gradually introducing non-convexity to reject outliers. This prevents premature convergence to undesirable local minima. As optimization progresses, μ gradually decays, allowing the robust kernel function to smoothly transition toward a non-convex form. This results in more aggressive outlier rejection during later stages of optimization.

We use a GNC variant of the Geman–McClure function for the optimization problem. The choice of the Geman–McClure function is driven by several advantages:

- Non-Convexity and Smoothness: The Geman–McClure function is a well-known non-convex robust kernel that suppresses large residuals more effectively than Huber or Cauchy functions. It also exhibits smoothness, which is essential for gradient-based optimization, avoiding discontinuities during the optimization process.
- Suitability for GNC: The Geman–McClure function is commonly used in GNC due to its analytical form and the ability to smoothly transition between convex and non-convex forms. By adjusting μ, it can effectively reduce the influence of large residuals (outliers) to nearly constant values, limiting their impact on the optimization.

The optimization problem is formulated as follows:

(10) $$\underset{x}{min}\sum_{i=1}^{N}\rho_{\mu }(∥e_{i}(x){∥}^{2})\;$$

where $\rho_{\mu }\left(r^{2}\right)$ is the Geman–McClure kernel function, defined as:

(11) $$\rho_{\mu }(r^{2})=\frac{\mu r^{2}}{\mu +r^{2}}\;\;$$

Here, the parameter μ controls the shape and degree of non-convexity of the kernel function. Specifically, μ determines the smooth transition from a convex to a non-convex form:

- When μ is large, $\rho_{\mu }(r^{2})$ approximates $r^{2}$, similar to least squares, which is suitable when few outliers are present.
- When μ is small, $\rho_{\mu }(r^{2})$ exhibits stronger suppression of outliers, reducing their influence on the optimization result.

Regarding the range of μ, it is a positive parameter typically chosen between (0,1]. Initially, μ is set to a relatively large value (e.g., $\mu^{\left(0\right)}=1$), at which point the kernel behaves similarly to least squares. As the optimization progresses, μ is gradually reduced, transitioning the kernel to a more non-convex form and increasing the suppression of outliers.

During the optimization, μ is decayed by a factor η∈(0,1) after each iteration to progressively increase the penalty on outliers. Therefore, μ starts large and decreases over time to improve outlier rejection. If μ is set too high, the algorithm may prematurely converge to a least-squares solution, failing to adequately reject outliers.

The iterative optimization process is as follows:

1. Initialization: The optimization begins with a large initial non-convexity parameter $\mu^{\left(0\right)}=1$. At this stage, the robust kernel approximates a quadratic function, starting from a convex region.
2. Weight Calculation: In the k-th iteration, a dynamic weight $w_{i}^{\left(k\right)}$ is calculated for each residual $e_{i}^{\left(k\right)}$. This weight is derived from the derivative of the Geman–McClure kernel function based on the current residual value and the non-convexity parameter $\mu^{\left(k\right)}$, reflecting the confidence of the current residual as an inlier or outlier:
  (12) $$w_{i}^{\left(k\right)}=\frac{{\left(\mu^{\left(k\right)}\right)}^{2}}{(\mu^{\left(k\right)}+∥e_{i}^{\left(k\right)}{∥}^{2}{)}^{2}}\;$$
3. Gauss–Newton System Solution: Using the calculated dynamic weights, the non-convex optimization problem is transformed into a weighted least-squares problem, which is then solved using the Gauss–Newton method:
  (13) $$(J^{T}W^{\left(k\right)}J)\Delta x=-J^{T}W^{\left(k\right)}e^{\left(k\right)}$$
  where J is the Jacobian matrix of the residuals with respect to the state vector, and $W^{\left(k\right)}$ is a diagonal matrix with $w_{i}^{\left(k\right)}$ as the diagonal elements.
4. State Update: The state vector is updated based on the solved step size:

(14) $$x^{\left(k+1\right)}=x^{\left(k\right)}+\Delta x$$

5 Non-Convexity Parameter Decay: After each iteration, μ is gradually reduced by a decay factor η∈(0,1):

(15) $$\mu^{\left(k+1\right)}=\eta \mu^{\left(k\right)}$$

This gradual decay ensures that the robust kernel function transitions from a near-quadratic form to a stronger non-convexity, increasing the penalty on outliers as optimization progresses. The iteration terminates when $\mu^{\left(k\right)}$ falls below a preset threshold ϵ.

The GNC optimization method avoids local minima by smoothly transitioning from a convex approximation to the original non-convex problem. This strategy greatly enhances system robustness, especially in scenarios with a high proportion of outliers. The GNC strategy is tightly integrated into the local Bundle Adjustment (BA) framework, enabling robust joint optimization of camera poses and map points (landmarks).

### 3.5. Point-Line Feature Fusion Frontend with XFeat and SOLD2

This section introduces a novel point-line feature fusion approach designed to address critical challenges in visual SLAM, including sparse point feature distribution in sparse texture areas and high mismatch rates in repetitive structures. By combining XFeat point features with SOLD2 line features, we create a multi-geometrically constrained visual frontend that enhances system robustness.

XFeat provides robust point feature extraction in texture-rich regions, whereas SOLD2 line features help maintain stability in texture-deprived environments. While this fusion frontend improves robustness, it comes with additional computational cost, and thus, it is implemented as an optional replacement for the standard SuperPoint + PLNet frontend in HiRo-SLAM.

The XFeat point feature extraction network uses a lightweight architecture, achieving exceptional feature repeatability. Through a hierarchical downsampling strategy, it effectively integrates multi-scale features, demonstrating strong scale invariance and providing precise local positioning in texture-rich regions. However, in weakly textured environments, the number of detectable point features significantly decreases, leading to inadequate visual constraints and a drop in performance.

To address this limitation, we integrate SOLD2 line features as complementary visual cues. Line segments are stable in texture-sparse environments, such as walls and floors, and offer superior discriminative power in repetitive structures. SOLD2 extracts precise line segments by jointly predicting junction heatmaps J and line heatmaps H.

For point feature learning, we employ a contrastive learning framework with a double Softmax loss:

(16) $$L_{\mathrm{point}}=-\sum_{i}log({\mathrm{softmax}}_{r}(S{)}_{ii})-\sum_{i}log({\mathrm{softmax}}_{r}(S^{⊤}{)}_{ii})$$

where S is the similarity matrix, with $S_{ij}$ denoting the descriptor similarity between points i and j, and r is the temperature parameter.

For line feature optimization, we use both detection and descriptor losses:

Junction detection is handled with a grid-based cross-entropy loss:

(17) $$L_{\mathrm{junc}}=\frac{64}{h\times w}\sum_{i,j=1}^{h/8,w/8}-\;log(\frac{exp(J_{ijy_{ij}}^{c})}{\sum_{k=1}^{65}exp(J_{ijk}^{c})})$$

where $J^{c}$ is the coarse-level junction feature map, and $y_{ij}$ represents ground-truth labels for accurate line endpoint localization.

Line heatmap prediction employs a binary cross-entropy loss:

(18) $$L_{\mathrm{line}}=\frac{1}{h\times w}\sum_{i,j=1}^{h,w}-H_{ij}^{GT}log(H_{ij})$$

where $H^{GT}$ is the binary ground-truth heatmap, optimizing line presence probability estimation.

Line descriptor learning adopts a triplet loss formulation:

(19) $$L_{\mathrm{desc}}=\frac{1}{n}\sum_{i=1}^{n}max(0,M+p_{i}-n_{i})$$

where $p_{i}$ and $n_{i}$ represent the distances to positive and hardest negative samples, respectively, with M being the margin hyperparameter, enhancing line descriptor discriminability.

We balance these objectives using a multi-task learning framework with learnable weights:

(20) $$L_{\mathrm{total}}=e^{-w_{\mathrm{junc}}}L_{\mathrm{junc}}+e^{-w_{\mathrm{line}}}L_{\mathrm{line}}+e^{-w_{\mathrm{desc}}}L_{\mathrm{desc}}+w_{\mathrm{junc}}+w_{\mathrm{line}}+w_{\mathrm{desc}}$$

where $w_{\mathrm{junc}}$, $w_{\mathrm{line}}$, $w_{\mathrm{desc}}$ are learnable weighting parameters.

The proposed fusion frontend combines the precise local constraints from point features with the global structural constraints from line features, significantly improving robustness in challenging scenarios, including weak textures and repetitive structures. At the same time, it maintains the computational efficiency necessary for real-time applications.

## 4. Experimental Results

This section presents a comprehensive evaluation of the proposed visual-inertial SLAM framework. All experiments were conducted on a computational platform equipped with an NVIDIA GeForce RTX 2080 Ti GPU, manufactured by NVIDIA Corporation, Santa Clara, CA, USA. Evaluation was conducted using visual-inertial sequences from the EuRoC MAV, TUM-VI, and OIVIO datasets. To ensure state estimation accuracy, our framework incorporates specific IMU noise models. For the EuRoC MAV sequences, we adopted the physical specifications of the industrial-grade ADIS16448 MEMS IMU. The noise densities for the gyroscope and accelerometer were set to $1.6968\times 10^{-4}\;rad/s/\sqrt{Hz}$ and $2.0000\times 10^{-3}\;m/s^{2}/\sqrt{Hz}$, respectively, while the corresponding random walk values were $1.9393\times 10^{-5}\;rad/s^{2}/\sqrt{Hz}$ and $3.0000\times 10^{-3}\;m/s^{3}/\sqrt{Hz}$. These parameters are critical for IMU pre-integration and subsequent global optimization within the SLAM backend. The platform integrates both camera and IMU data, with time synchronization achieved during calibration. The Absolute Trajectory Error (ATE) [35] is used as the primary evaluation metric. Trajectory alignment is performed through Sim(3) transformation between the estimated and ground-truth trajectories, with quantitative assessment conducted using root mean square error (RMSE), as well as mean, median, and standard deviation (S.D.) statistics.

We evaluate HiRo-SLAM on three benchmark datasets: the EuRoC MAV dataset [36] and the TUM-VI dataset [37], and the OIVIO dataset [38]. The EuRoC MAV dataset provides millimeter-level ground truth trajectories recorded by high-precision motion capture systems (e.g., Vicon or laser tracking). These trajectories serve as a reliable reference for performance evaluation. The dataset includes sequences of varying difficulty: easy sequences feature smooth motion in richly textured environments; medium sequences introduce moderate challenges such as camera shake and sudden illumination changes; and difficult sequences involve rapid motion, large texture-less areas, and severe lighting variations. These challenging conditions test the algorithm’s ability to handle both visual and inertial uncertainties. The ground truth accuracy, with errors well below typical visual odometry ranges, provides a solid benchmark for comparison.

Similarly, the TUM-VI dataset offers high-precision ground truth data, recorded using motion capture systems like Vicon, ensuring millimeter-level accuracy. It includes sequences captured under extreme conditions, such as rapid motion, smooth surfaces, and significant illumination variations. Sequences like room5 and room6 are particularly challenging, featuring motion blur and visual degradation, making them ideal for testing SLAM systems under real-world stress. The ground truth accuracy, also in the millimeter range, provides a reliable benchmark for evaluating SLAM performance in demanding environments with rapid motion and lighting changes.

The OIVIO dataset is designed to evaluate SLAM algorithms in dark, low-light environments with controlled illumination. It includes 36 sequences recorded in mines and tunnels, with a total of over 145 min of stereo camera and IMU data. Each sequence is illuminated by an onboard light, with intensity levels set to 1350, 4500, or 9000 lumens, corresponding to different test conditions. The dataset provides ground truth data with millimeter-level accuracy, allowing for robust performance evaluation under various challenging conditions, such as fluctuating light and high-speed motion. This dataset provides an ideal scenario for testing SLAM systems under real-world illumination constraints, making it an important addition to the evaluation.

We compare the performance of HiRo-SLAM with state-of-the-art methods, highlighting its superior effectiveness. Additionally, ablation experiments quantitatively evaluate the contribution of each algorithmic components to the overall system accuracy.

### 4.1. Evaluation on the EuRoc Dataset

This section presents a systematic evaluation of HiRo-SLAM’s performance using the EuRoC dataset.

As detailed in Table 1, HiRo-SLAM outperforms competing methods, securing the lowest RMSE in 9 of 11 sequences. Compared to the second-best system, HiRo-SLAM reduces the average translational error by approximately 30.0%. When compared to well-established methods like ORB-SLAM3, HiRo-SLAM achieves optimal performance in 10 sequences, reducing the average translational error by about 40.0%.

Notably, in the MH_02_easy sequence, HiRo-SLAM’s RMSE is 0.022 m, which is slightly higher than the best-performing method, which achieved 0.013 m. The MH_02_easy sequence is classified as “easy,” featuring smooth motion and rich textures. In such scenarios, some baseline methods may already extract sufficiently stable features, which, along with their simpler models, provide a slight advantage due to lower computational overhead. Nonetheless, HiRo-SLAM still surpasses most other baseline methods in this sequence and demonstrates significant improvements in overall performance.

In more complex sequences with challenging conditions, such as V2_03_difficult, HiRo-SLAM maintains robust pose estimation accuracy, especially in scenarios involving rapid motion and large rotations. These results validate the effectiveness of HiRo-SLAM’s key innovations: Precise Camera Projection Modeling (PCPM), P-ANMS feature selection, and GNC-based robust optimization. PCPM, by embedding a fully differentiable camera model with complete distortion parameters into nonlinear optimization, ensures accurate handling of camera intrinsics and distortion. This effectively prevents systematic error accumulation, thereby maintaining high accuracy in long-term geometric estimation. P-ANMS, by optimizing feature distribution, provides uniform and strong visual constraints, even in complex environments. GNC ensures reliable optimization even in the presence of outliers, which is critical in scenarios involving significant visual degradation or dynamic changes.

Table 2 presents the standard deviation (S.D.) of translational error for various sequences from the EuRoC dataset. While RMSE provides a general measure of accuracy, the standard deviation adds valuable insight into the variability and dispersion of the error over time. This metric helps assess the consistency of the localization performance, particularly in sequences with varying motion dynamics and environmental challenges.

While RMSE reflects overall accuracy, the S.D. represents the stability and jitter of the estimation, which is a key component of the error budget in VINS.

In terms of overall performance, HiRo-SLAM demonstrates a clear advantage with an average S.D. of 0.010, significantly lower than that of ORB-SLAM3 (0.018) and AirSLAM (0.016). This indicates that HiRo-SLAM exhibits lower error variability, reflecting superior stability and consistency across all test sequences. Notably, in more challenging sequences such as V2_03_difficult, HiRo-SLAM continues to outperform the other methods, maintaining smaller fluctuations in error.

The inclusion of the standard deviation further highlights HiRo-SLAM’s robustness, particularly in dynamic environments and areas with texture-less regions, where traditional methods exhibit larger error fluctuations.

### 4.2. Trajectory Visualization

HiRo-SLAM is evaluated across three sequences from the EuRoC MAV dataset, each presenting unique challenges. The V1_01_easy sequence features smooth motion in a richly textured environment with stable lighting, providing a baseline for performance under ideal conditions. The V2_02_medium sequence introduces moderate challenges, such as slight camera shake and fluctuating lighting, requiring more robust handling. In the most demanding V2_03_difficult sequence, rapid motion, large texture-less areas, and severe lighting changes test the system’s ability to maintain accuracy under stress. These conditions, reflecting a range of real-world scenarios, highlight HiRo-SLAM’s robustness in handling visual and inertial uncertainties.

**Figure 3:**
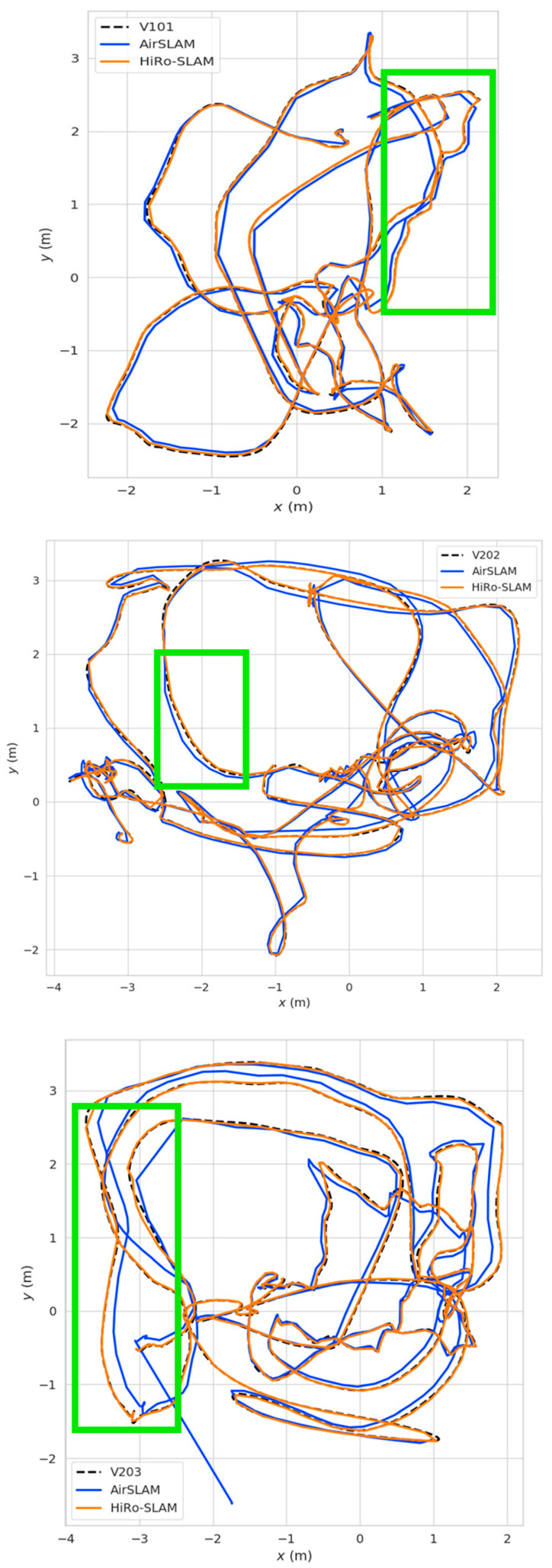
Comparison of tracking performance for AirSLAM and HiRo-SLAM on the EuRoC dataset. The dotted line represents the ground truth trajectory. The green rectangles highlight regions of superior performance for HiRo-SLAM compared to AirSLAM.

Figure 3 presents a comparative visualization of the estimated trajectories from HiRo-SLAM and the second-best method across the three sequences. This visualization highlights HiRo-SLAM’s superior performance, particularly in suppressing drift and maintaining accurate alignment with the ground truth in challenging sequences.

In the V1_01_easy sequence, both HiRo-SLAM and AirSLAM should align closely with the ground truth due to the stable, well-textured environment. However, in the green-highlighted region, HiRo-SLAM (blue line) consistently stays closer to the ground truth than AirSLAM (orange line), highlighting its superior localization performance even under ideal conditions.

In the V2_02_medium sequence, HiRo-SLAM continues to track the ground truth well despite moderate shake and lighting fluctuations. In contrast, AirSLAM shows noticeable deviations. The green-highlighted region illustrates where AirSLAM starts to drift more than HiRo-SLAM, emphasizing HiRo-SLAM’s robustness in maintaining localization accuracy in moderate challenging conditions.

In the V2_03_difficult sequence, the performance gap between HiRo-SLAM and AirSLAM widens significantly. HiRo-SLAM (blue line) remains closely aligned with the ground truth throughout the entire motion sequence, even in areas with high frame dropout, motion blur, and texture-less regions. On the other hand, AirSLAM’s trajectory (orange line) diverges more, particularly in areas with motion blur or difficult scenes, demonstrating noticeable drift accumulation. This demonstrates HiRo-SLAM’s ability to effectively handle challenging environmental factors, making it more reliable in real-world scenarios.

These qualitative observations align with the quantitative ATE metrics in Section 4.1, collectively confirming that HiRo-SLAM outperforms in suppressing cumulative errors and maintaining long-term accuracy.

The particularly notable improvement in accuracy on challenging sequences like V2_03_difficult directly validates the effectiveness of our Precise Camera Projection Modeling (PCPM) strategy. By precisely integrating analytical Jacobians with complete distortion parameters into the optimization, our system dynamically corrects model errors, ensuring long-term geometric estimation precision. Additionally, P-ANMS ensures uniform feature distribution and stable tracking during complex movements, while GNC robust optimization mitigates the impact of potential outliers during aggressive motion, enhancing the system’s robustness in challenging scenarios.

**Figure 4:**
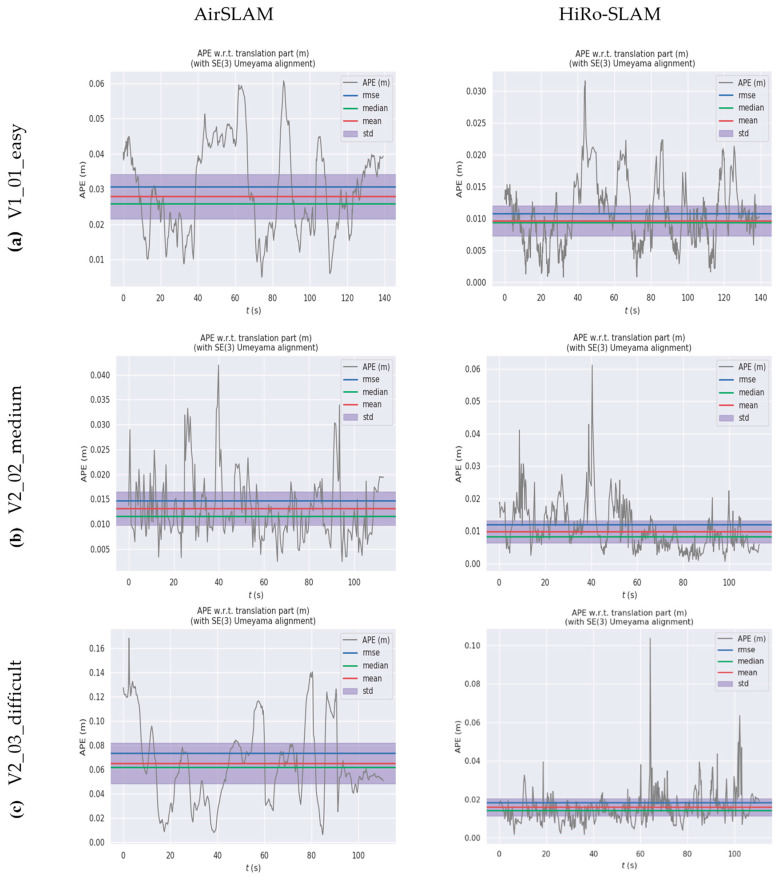
Comparison of ATE over time for AirSLAM and HiRo-SLAM on the EuRoC dataset.

Figure 4 further complements this analysis by showing the temporal evolution of ATE for both methods across the three sequences.

As illustrated in Figure 4, HiRo-SLAM consistently yields significantly lower ATE compared to AirSLAM across the V1_01_easy, V2_02_medium, and V2_03_difficult sequences. In terms of mean values (red lines), medians (green lines), and standard deviations (purple shaded areas), HiRo-SLAM exhibits reduced fluctuations and remains closer to zero.

In the highly challenging V2_03_difficult scenario, AirSLAM’s APE peaks near 0.16 m, whereas HiRo-SLAM maintains a peak of approximately 0.1 m with errors densely clustered in the lower range. These results demonstrate that HiRo-SLAM outperforms AirSLAM in both estimation accuracy and robustness across diverse environments.

### 4.3. Evaluation on the TUM-VI Dataset

To evaluate the system’s performance under complex fisheye lens distortion scenarios, we conducted tests on the TUM-VI dataset.

As shown in Table 3, HiRo-SLAM significantly improves pose estimation performance over existing methods by introducing Precise Camera Projection Modeling (PCPM). The experimental results demonstrate that HiRo-SLAM reduces the mean reprojection error by 10% compared to ORB-SLAM3 across multiple sequences in the TUM-VI dataset, validating the effectiveness of our precise camera projection strategy. Notably, even in highly distorted peripheral regions of fisheye images, HiRo-SLAM maintains stable feature tracking and accurate pose estimation, confirming its robustness and adaptability in challenging imaging conditions.

### 4.4. Evaluation on the OIVIO Dataset

This section presents a systematic evaluation of HiRo-SLAM’s performance using the OIVIO dataset.

As shown in Table 4, the evaluation of HiRo-SLAM on the OIVIO dataset demonstrates its competitive performance across various sequences. HiRo-SLAM consistently achieves low translational error, with an average RMSE of 0.056 m, outperforming many state-of-the-art systems. In particular, it shows superior accuracy compared to PL-SLAM, Basalt, and ORB-SLAM3, particularly in more challenging sequences like MN_015_GV_01 and MN_015_GV_02, where HiRo-SLAM’s error is notably lower. The system performs comparably to AirSLAM but with slight advantages in most sequences. These results indicate HiRo-SLAM’s robustness and effectiveness in handling diverse environments within the OIVIO dataset.

### 4.5. Ablation Studies

To quantitatively evaluate the individual contributions of our innovations, we conducted systematic ablation experiments on the EuRoC dataset, assessing each module’s performance across both standard and challenging scenarios. Table 5 summarizes the results from all sequences.

The ablation experiments reveal that HiRo-SLAM achieves a 31.15% improvement over the baseline system (SuperPoint + PLNet). This improvement can be attributed to the combined effects of our proposed innovations. Specifically, the Precise Camera Projection Model (PCPM) contributes 19.67% of the total gain, while the P-ANMS feature selection mechanism contributes 10.82%, and the GNC optimization strategy provides the remaining 0.66%.

Specifically, PCPM leads to a significant performance boost, with the average RMSE reduced from 0.0305 to 0.0245. This demonstrates the critical role of PCPM in effectively incorporating gradient information for camera intrinsics and distortion parameters into nonlinear optimization, which prevents the accumulation of systematic errors.

The introduction of P-ANMS further enhances performance, reducing the average RMSE to 0.0212. This confirms that P-ANMS, by optimizing the global spatial distribution of feature points, strengthens the geometric constraints, resulting in more robust pose estimation, particularly in weakly textured or unevenly distributed feature regions.

The full HiRo-SLAM system, incorporating PCPM, P-ANMS, and GNC-based robust optimization, achieves an average RMSE of 0.0210, very close to the version with just PCPM and P-ANMS. Although the direct contribution of GNC appears modest (0.66%), its role in ensuring system stability by mitigating the influence of outliers is crucial, especially in challenging scenarios like the room6 sequence in the TUM-VI dataset.

The combination of these innovations significantly enhances the overall accuracy and robustness of the system, particularly in demanding operational conditions.

### 4.6. Ablation Studies on P-ANMS

For this study, the parameters are set as follows: the keypoint detection threshold is 0.002, and the visibility pyramid consists of 6 levels. To assess the robustness of the method, an ablation study was conducted to evaluate the sensitivity of the system to these parameters on V1_02_medium, as shown in Table 6.

The ablation results show the trade-offs in terms of system accuracy, robustness, and efficiency. The experiments indicate that the system is relatively insensitive to moderate changes in these parameters, showcasing its robustness.

### 4.7. Evaluation of the Point-Line Feature Fusion Frontend

To validate the effectiveness of our point-line feature fusion strategy, we conducted comparative experiments on the EuRoC dataset, evaluating the baseline system (SuperPoint + PLNet) against a variant that incorporates XFeat + SOLD2. The results are summarized in Table 7.

Table 7 shows that the XFeat + SOLD2 frontend improves pose estimation accuracy in the challenging V2_02 sequence, reducing the RMSE from 0.018 m to 0.0147 m. This represents an 18.33% improvement over the baseline (SuperPoint + PLNet), highlighting the enhanced robustness of the hybrid feature representation. The improvement is particularly noticeable in scenarios with weak textures or rapid motion, where traditional point-based features tend to degrade.

## 5. Conclusions

This paper presents HiRo-SLAM, a visual-inertial SLAM framework that offers significant improvements in accuracy and robustness. The system’s performance improvements stem from four key contributions: First, Precise Camera Projection Modeling (PCPM) integrates a fully differentiable camera model with distortion parameters into nonlinear optimization, ensuring precise gradient computation and mitigating systematic errors. Second, Visibility Pyramid-based Adaptive Non-Maximum Suppression (P-ANMS) optimizes feature distribution by quantifying the contribution of candidate points to global spatial uniformity, enhancing visual constraints. Third, Graduated Non-Convexity (GNC) applies a non-convex cost function with dynamic weighting to suppress outliers, improving convergence and estimation reliability. Finally, Lightweight Point-Line Feature Fusion combines XFeat point features with SOLD2 line features, improving perceptual capabilities and feature tracking in challenging environments.

Experimental evaluations demonstrate that HiRo-SLAM outperforms current state-of-the-art methods across multiple benchmarks, particularly in dynamic environments with significant illumination changes. HiRo-SLAM reduces the average absolute trajectory error (ATE) by over 30.0% compared to advanced methods, achieving millimeter-level accuracy on specific sequences, and delivering state-of-the-art (SOTA) performance.

HiRo-SLAM’s innovations not only demonstrate superior performance on benchmark datasets but also offer significant advantages in real-world applications. Its high accuracy and robustness make it particularly suitable for challenging environments where current VINS methods struggle. HiRo-SLAM ensures long-term pose estimation accuracy, even in the presence of significant optical distortions. Such high precision makes it ideal for applications like autonomous drone navigation in complex urban environments. The Visibility Pyramid-based Adaptive Non-Maximum Suppression (P-ANMS) improves feature distribution in feature-sparse or repetitive environments, significantly enhancing the reliability and stability of AR/VR systems in low-texture spaces. Graduated Non-Convexity (GNC) enhances robustness to dynamic outliers, making HiRo-SLAM effective for mobile robotics in dynamic and cluttered environments, such as warehouses or factories, where traditional systems often fail. The Point-Line Feature Fusion Frontend improves stability in environments with weak textures or repetitive structures, which is particularly useful for long-term operations in challenging scenarios. These innovations allow HiRo-SLAM to overcome the limitations of traditional VINS systems in highly dynamic or texture-deprived environments.

However, certain limitations should be noted. While the core system using SuperPoint + PLNet excels in well-textured environments, its performance may degrade in extremely texture-deprived or unstructured scenarios, where traditional point-based systems still perform competitively. Additionally, while the XFeat + SOLD2 fusion frontend improves robustness in environments with weak textures or repetitive structures, it does not completely eliminate challenges in highly unstructured or featureless areas. These findings indicate that further work is needed to optimize HiRo-SLAM’s performance in such challenging environments. Future research will focus on improving the balance between estimation accuracy and computational efficiency, particularly in texture-deprived or highly dynamic settings.

## Figures and Tables

**Table 1:** Translational error (RMSE) on the EuRoc dataset (unit: m). The best results are in bold.

Sequence	OpenVINS ^1^	PL-SLAM ^1^	Basalt ^1^	ORB-SLAM3 ^1^	AirSLAM ^1^	HiRo-SLAM ^2^
MH_01_easy	0.072	0.042	0.080	0.036	0.019	**0.015**
MH_02_easy	0.143	0.052	0.060	0.033	**0.013**	0.022
MH_03_medium	0.086	0.040	0.050	0.035	0.025	**0.023**
MH_04_difficult	0.173	0.064	0.100	**0.051**	0.056	**0.051**
MH_05_difficult	0.247	0.070	**0.040**	0.082	0.051	0.045
V1_01_easy	0.055	0.042	0.020	0.038	0.032	**0.011**
V1_02_medium	0.060	0.046	0.030	0.014	0.014	**0.009**
V1_03_difficult	0.059	0.069	0.030	0.024	0.025	**0.018**
V2_01_easy	0.054	0.061	0.020	0.032	0.014	**0.008**
V2_02_medium	0.047	0.057	0.059	0.014	0.018	**0.012**
V2_03_difficult	0.141	0.126	0.052	0.024	0.068	**0.018**
Average	0.103	0.061	0.049	0.035	0.030	**0.021**

^1^ results taken from [8]. ^2^ computed by the authors using the official evaluation tools.

**Table 2:** Translational error (S.D.) on the EuRoc dataset (unit: m). The best results are in bold.

Sequence	ORB-SLAM3 ^1^	AirSLAM ^1^	HiRo-SLAM ^1^
MH_01_easy	0.020	0.011	**0.005**
MH_02_easy	0.013	**0.005**	0.008
MH_03_medium	0.018	**0.010**	0.013
MH_04_difficult	0.027	0.029	**0.02** **4**
MH_05_difficult	0.027	0.024	**0.02** **1**
V1_01_easy	0.023	0.019	**0.00** **5**
V1_02_medium	0.019	0.019	**0.00** **4**
V1_03_difficult	0.012	0.011	**0.009**
V2_01_easy	0.019	0.005	**0.00** **4**
V2_02_medium	**0.005**	0.010	0.007
V2_03_difficult	0.010	0.028	**0.009**
Average	0.018	0.016	**0.010**

^1^ computed by the authors using the official evaluation tools.

**Table 3:** Translational error (RMSE) on the TUM-VI dataset (unit: m). The best results are in bold.

Dataset	AirSLAM ^2^	Basalt ^1^	OKVIS ^1^	DM-VIO ^1^	OpenVINS ^1^	ORB-SLAM3 ^3^	HiRo-SLAM ^2^
room1	0.100	0.090	0.060	0.030	0.062	0.010	**0.008**
room2	0.103	0.070	0.110	0.130	0.093	0.010	**0.007**
room3	0.069	0.130	0.070	0.090	0.079	0.010	**0.014**
room4	0.094	0.050	0.030	0.040	0.027	0.010	**0.009**
room5	0.095	0.130	0.070	0.060	0.074	0.010	**0.007**
room6	0.766	0.020	0.040	0.020	0.020	0.010	**0.007**
Average	0.205	0.082	0.063	0.062	0.059	0.010	**0.009**

^1^ results taken from [39]. ^2^ computed by the authors using the official evaluation tools. ^3^ results taken from [2].

**Table 4:** Translational error (RMSE) on the OIVIO dataset (unit: m). The best results are in bold.

Sequence	PL-SLAM ^1^	Basalt ^1^	ORB-SLAM3 ^1^	AirSLAM ^1^	HiRo-SLAM ^2^
MN_015_GV_01	1.238	0.216	0.066	0.054	**0.046**
MN_015_GV_02	0.853	0.153	0.069	0.052	**0.048**
MN_050_GV_01	1.143	0.186	0.063	**0.062**	**0.062**
MN_050_GV_02	0.921	0.103	0.053	0.048	**0.046**
MN_100_GV_01	0.831	**0.197**	**0.051**	0.064	0.058
MN_100_GV_02	0.609	0.092	0.063	**0.042**	0.050
TN_015_GV_01	1.579	0.148	**0.053**	0.057	**0.053**
TN_050_GV_01	1.736	0.521	0.082	**0.065**	0.068
TN_100_GV_01	1.312	0.116	0.086	0.078	**0.077**
Average	1.358	0.192	0.065	0.058	**0.056**

^1^ results taken from [8]. ^2^ computed by the authors using the official evaluation tools.

**Table 5:** Ablation experiments on the EuRoC dataset (unit: m, four decimal places due to small error values). The best results are in bold.

Sequence	Baseline (SuperPoint + PLNet)	Baseline + PCPM	Baseline +PCPM + P-ANMS	Baseline +PCPM + P-ANMS + GNC (HiRo-SLAM)
MH_01_easy	0.0191	0.0175	**0.0097**	0.0155
MH_02_easy	**0.0128**	0.0256	0.0288	0.0220
MH_03_medium	0.0252	**0.0210**	0.0276	0.0231
MH_04_difficult	0.0564	0.0666	**0.0411**	0.0512
MH_05_difficult	0.0508	**0.0445**	0.0456	0.0446
V1_01_easy	0.0323	0.0115	0.0125	**0.0108**
V1_02_medium	0.0142	0.0088	0.0087	**0.00** **77**
V1_03_difficult	0.0249	0.0161	**0.0155**	0.0178
V2_01_easy	0.0141	0.0087	0.0092	**0.0079**
V2_02_medium	0.0177	0.0127	**0.0094**	0.0120
V2_03_difficult	0.0681	0.0366	0.0253	**0.0184**
Average	0.0305	0.0245	0.0212	**0.021** **0**
Percentage Improvement (%) ^1^	-	19.67	30.49	**3** **1.15**

^1^ The Percentage Improvement row shows the performance improvement of the proposed method relative to the baseline (Super-Point + PLNet), expressed as a percentage.

**Table 6:** Translational error (RMSE) of the ablation experiments on V1_02_medium (unit: m). Each configuration evaluates the sensitivity of HiRo-SLAM to the key parameters used in the feature selection process. The best results are in bold.

Pyramid Levels	Keypoint Threshold	RMSE
0	0.002	0.0097
0	0.004	0.0096
0	0.006	0.0098
3	0.002	0.0096
3	0.004	0.0094
3	0.006	0.0095
**6**	**0.002**	**0.0090**
6	0.004	0.0093
6	0.006	0.0094
8	0.002	0.0096
8	0.004	0.0097
8	0.006	0.0097

**Table 7:** Results Comparison of the Feature Frontend (SuperPoint + PLNet) with the Point-Line Feature Fusion Frontend (XFeat + SOLD2) on ATE (unit: m) for the V2_02 sequence. The best results are in bold.

Methods	V2_02			
	RMSE	Mean	Median	S.D.
SuperPoint + PLNet	0.0180	0.0151	0.0129	0.0095
XFeat + SOLD2	**0.0147**	**0.0131**	**0.0116**	**0.0067**
Percentage Improvement (%)	18.33	13.25	10.08	29.47

## Data Availability

No new data were created in this study. All datasets analyzed are publicly available from their original sources [EuRoc, TUM-VI, OIVIO].
